# Deployment Optimization Algorithms in Wireless Sensor Networks for Smart Cities: A Systematic Mapping Study

**DOI:** 10.3390/s22145094

**Published:** 2022-07-07

**Authors:** Huda M. Abdulwahid, Alok Mishra

**Affiliations:** 1 Department of Modeling and Design of Engineering Systems (MODES), Atilim University, Ankara 06830, Turkey; abdulwahid.hudamkhaled@student.atilim.edu.tr; 2 Department of Software Engineering, Atilim University, Ankara 06830, Turkey; 3 College of Administration and Economic, Anbar University, Ramadi 31001, Iraq; 4 Informatics and Digitalization Group, Molde University College—Specialized University in Logistics, 6410 Molde, Norway

**Keywords:** smart city, wireless sensor network (WSN), deployment, coverage, connectivity, meta-heuristic

## Abstract

In recent years, different types of monitoring systems have been designed for various applications, in order to turn the urban environments into smart cities. Most of these systems consist of wireless sensor networks (WSN)s, and the designing of these systems has faced many problems. The first and most important problem is sensor node deployment. The main function of WSNs is to gather the required information, process it, and send it to remote places. A large number of sensor nodes were deployed in the monitored area, so finding the best deployment algorithm that achieves maximum coverage and connectivity with the minimum number of sensor nodes is the significant point of the research. This paper provides a systematic mapping study that includes the latest recent studies, which are focused on solving the deployment problem using optimization algorithms, especially heuristic and meta-heuristic algorithms in the period (2015–2022). It was found that 35% of these studies updated the swarm optimization algorithms to solve the deployment problem. This paper will be helpful for the practitioners and researchers, in order to work out new algorithms and seek objectives for the sensor deployment. A comparison table is provided, and the basic concepts of a smart city and WSNs are presented. Finally, an overview of the challenges and open issues are illustrated.

## 1. Introduction

**Figure 1:**
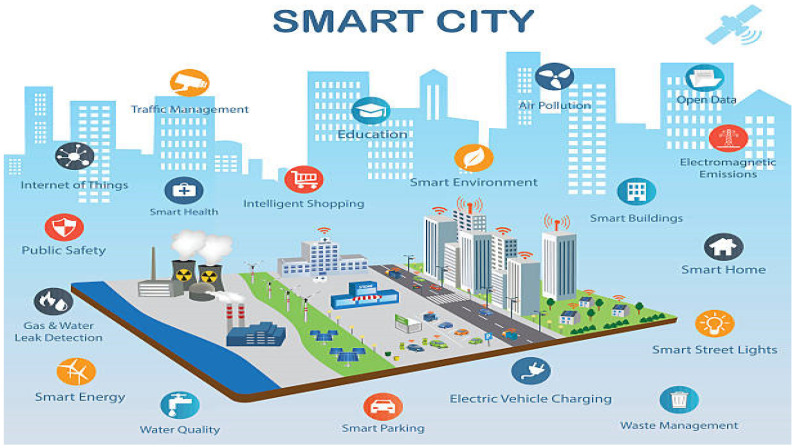
Smart city applications [1].

Many large cities around the world are adjusting to become smart cities, in order to enhance the quality of life for their citizens. With the use of digital, information, and communication technologies, traditional networks and services have become more effective. It means smarter transport networks and more efficient water supply, waste disposable facilities, and upgraded ways to heat and light buildings; as shown in Figure 1, it provides a city administration that is more interactive and responsive. It also enhances the use of city infrastructures and resources.

The basic component of smart city monitoring systems is wireless sensor networks, which consist of a large number of sensor nodes that are used for collecting and processing data. Due to the small size and low cost of sensor nodes, different WSNs are used for different applications, and this plays a vital role in the existence of Internet of Things (IoT) technology, which facilitates the building of a smart city, since it comes up with timely information that helps in deciding-making regarding comfort or safety. WSNs always comes up with new applications that enrich the IoT vision. Therefore, there is always a need for advanced and updated models and strategies for supporting on-demand WSN deployment to meet countries’ goals in increasing surveillance, intelligence, and reconnaissance in many safety-critical applications, where the IoT will also have an important role.

Though designing WSN many challenges can appear in the form of deployment, localization, communication, data gathering, coverage, and tracking, the most important issue that we focus on here in this research is the deployment problem, which is the first major challenge in designing a WSN monitoring system for a smart application.

**Motivations:** Most of the research in the literature focused on the deployment problem of WSNs in general. Based on the idea of monitoring large and open spaces, on the other hand, very few studies have been conducted regarding this problem in smart cities for reasons that are listed as follows [2]:

- The monitored area: some areas are large and require a network structure, such as water distribution networks, transportation networks, and streetlight networks. Some areas required three-dimensional (3D) monitoring, such as the structural health monitoring of bridges and towers.
- Different types of sensor nodes for the same measurements: static inductive loops or static cameras, for example, can be used to measure the traffic volume on the roads.
- Densely deployed sensor network in the monitored area: different sensor nodes for different applications exist in the same monitoring area.

Due to these reasons, some challenges have appeared, which affect the process of sensing in smart city monitoring systems, the most significant of which is the deployment of sensor nodes, which is limited by the monitored area’s structure, that is, what kind of sensor nodes are required and where they should be deployed to satisfy the best coverage, connectivity, and cost-effective monitoring.

**Contributions:** This research study presents a systematic mapping study that will focus on the node deployment problem for smart city monitoring systems over the period 2015–2022 by using meta-heuristic optimization algorithms. Therefore, this research aims to answer the research questions that are related to this problem and, in that way, could be helpful for researchers and practitioners/developers to better understand the deployment problem in that field. This will facilitate finding new solutions and adding further contributions to its development.

This paper is organized as follows: Section 2 describes the background of infrastructures and technologies that support the smart city and WSN, as well as the preliminaries of WSN deployment. Section 3 includes the literature review on the studies that discussed the deployment algorithms of WSN for different applications. In Section 4, the research method is illustrated. In Section 5, the results of the research questions are obtained and the open-research issues for WSN deployment are illustrated. Finally, Section 6 concludes the paper.

## 2. Background

### 2.1. Smart City

**Figure 2:**
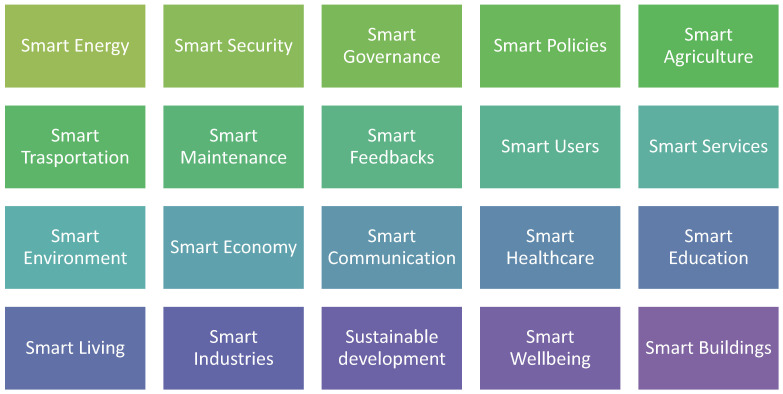
Smart city subsystems.

It is expected that 66% of the world’s population will live in urban areas by 2050. Providing this population with adequate resources, including sufficient energy, safe food, and clean water, with economic and social guarantees and environmental sustainability is a real challenge. Many cities today hope to become the smart cities of tomorrow. In order to achieve this goal, a complex plan should be developed, including participation from public and private sectors, product vendors, and providers of information technology services [3,4]. Most of the smart city sub-systems are shown in Figure 2 [5].

To implement these applications and operations, many advanced technologies are used, and they are summarized as follows [6]:

1. **Wireless Sensor Network (WSN):** An essential component in smart cities that can be used to provide remote control and real-time monitoring for smart city resources and infrastructure conditions.
2. **Internet of Things (IoT):** The technology that facilitates the lifestyle of humans through connecting physical things with sensory devices and allowing them to interact between each other and with people.
3. **Cyber-Physical System (CPS):** Used to provide the connection between computation, networking, and physical processes; in other words, it is the umbrella that includes the interaction between the virtual and physical worlds.
4. **Robotics and Unmanned Aerial Vehicles (UAVs):** Support smart cities with useful automated services, such as environmental monitoring, traffic monitoring, telecommunication services, security and safety control, and enhanced delivery of services.
5. **Fog computing:** This technology aims to support low latency, location awareness, better mobility, synchronization, coordination, data streaming, and real-time services for smart city applications when there is a large distance between the cloud platform and smart city sensors and devices, as well as when there is a large number of heterogeneous sensors and devices distributed in large areas. This makes it difficult for cloud computing to manage and deal with this situation. So, in this case, fog computing is preferred.
6. **Cloud computing:** This technology represents an important element of any smart city system, since it provides scalable processing power, as well as cost-effective, large, and scalable data storage and updated software services that support, manage, and control different smart city applications.
7. **Big Data**: The collected sensory data will be analyzed using this technology, in order to support optimized decision-making for smart city applications.

### 2.2. Wireless Sensor Network Components and Node Architecture

**Figure 3:**
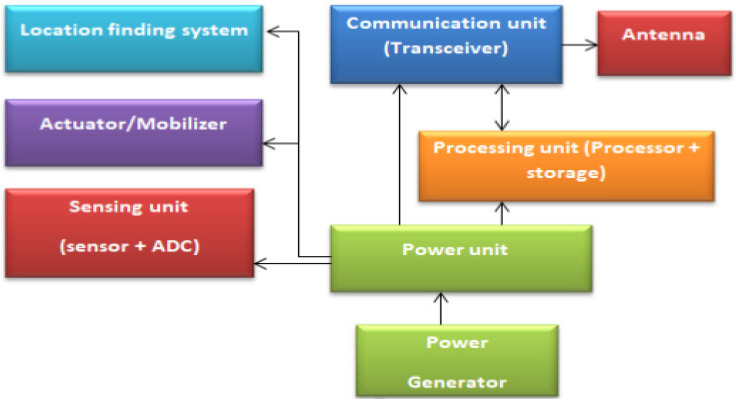
Sensor node architecture.

A WSN is composed of a large number of sensor nodes distributed over an area. These nodes are small in size and have a low cost, with limited storage capacity, processing capability, and energy. The sensor node architecture can be seen in Figure 3, which consists of the sensing unit that can be either the sensor or actuator, which senses the environment and take measurements of things such as temperature, humidity, sound, or vibration, thereby converting the collected analog signals into digital signals through the analog to digital converter. Then, it sends these signals or measurements to the processing unit to be processed and stored until sent through the communication unit to a relay node or a sink node [7,8].

All the parts of the sensor node require energy to operate, and the power unit can be either a battery, such as a CR2032, or some type of renewable energy, such as solar energy. This power unit should be stable and stay as long as possible, since the sensor nodes may be thrown in hazardous, rough, or harsh areas that cannot be reached for a replacement and where recharging the batteries is not possible. Another part is the communication unit, which is a radio transceiver unit that transmits and receives signals from one node to another through wireless channels. In the processing unit, the central processing unit (CPU) calculates the computational capabilities and energy consumption. The memory stores the required programs and data. Finally, if the sensor node is a mobile node, then it should have a location finding system, in order to get the required position, for example, the global positioning system (GPS).

The WSN consists of all, or parts, of the following components [9]:

1. Sensor node: A small-sized, low-powered node responsible for collecting data, processing it, and sharing it with other required nodes in the network.
2. Relay node: This node is used to communicate with the neighboring node as a midway node. This node is used to improve network reliability. It does not have any sensing or controlling processes.
3. Actor node: A high-end node used to set up and implement a decision based on the application’s demands. Usually, these nodes are resource-rich devices that are supplied with higher transmission power, processing capabilities, and battery life.
4. Cluster head: This node is used for gathering data from sensor nodes in WSN. There may be one or more inside the cluster, depending on the application’s requirements. This node should have high bandwidth and be reliable and secure.
5. Gateway node: This node is used to provide the connection between the WSN and outside networks
6. Sink node or base station: A control center where users can retrieve data gathered from the sensor network.

When deploying a huge number of sensor nodes that work together to monitor a specified target or a physical environment, the networking of these nodes is evenly significant, since the sensor nodes connect with each other, as well as with the base station, through wireless communications.

The base station sends the required tasks to the sensor nodes, while the latter gathers the requested information and sends it back to the BS for more processing. The BS sometimes acts as a gateway that sends the necessary data to the end-user through other networks. There are two types of network architectures, i.e., single- and multi-hop. In a single hop, each sensor node has a direct connection to the BS or sink node.

Although transmission is possible for long distances, the energy consumption is very high for communication, compared with the processing or data gathering. So, the multi-hop architecture is preferred, since the data can be transmitted over one or more intermediate nodes. Multi-hop architecture or clustering can be implemented in five ways, which are: hierarchal, partition-based, spectral, grid-based, and density-based clustering.

The hierarchal architecture depends on top-down or bottom-up approaches; it uses the tree structure, provides flexibility, and is preferred for point-to-point communication. In the partition-based method, the clusters are split into more sections; each section represents a cluster and can be used for a small number of nodes. The spectral type, used in the similarity matrix, needs low processing time, utilizes image processing, and is more suitable for a small number of nodes. In grid-based clustering, the area is divided into sections, depending on certain criteria, and the sensor nodes are added to these sections. This needs low processing time and computational complexity and provides high-performance data distribution. In the density-based method, the area with a larger number of clusters is called the high-density area, and it is separated from the low-density area, used with dynamic clustering, and provides better performance in a harsh environment [10].

### 2.3. Wireless Sensor Network Application

**Figure 4:**
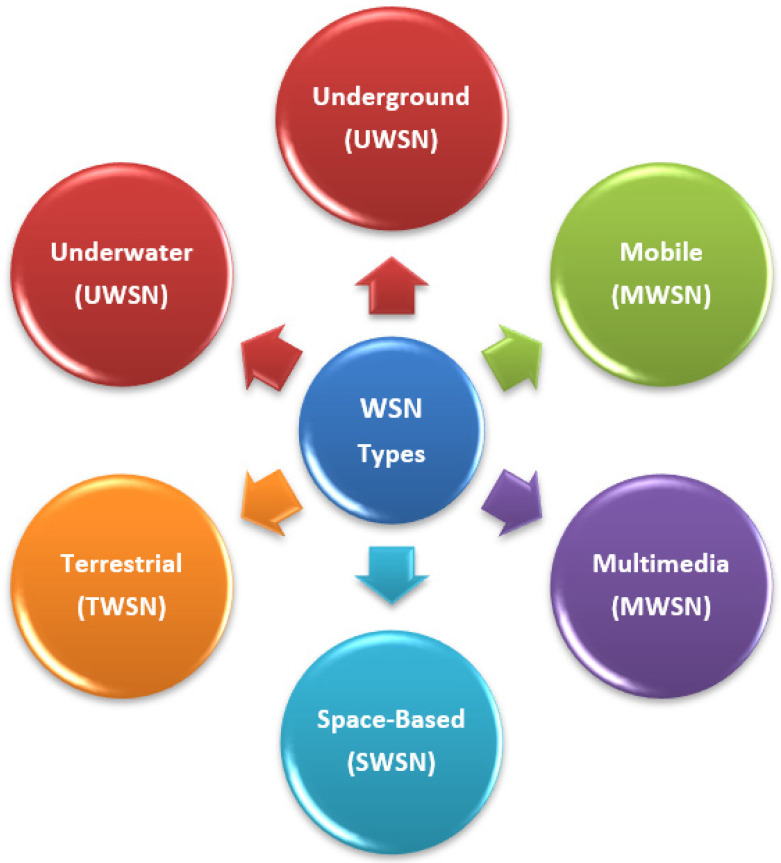
Types of WSNs.

Different types of WSN are used for different applications, as shown in Figure 4, the first use of WSNs was for military applications, where a system of an acoustic sensor on the ocean bottom was developed for monitoring the soviet submarines in the cold war; later on, in 1980, the work started on developing distributed sensor network (DSN). Now, WSNs are used in different areas, such as agriculture, bio-medical, health, traffic, industry, environment, and so on [7,11]. The work of WSN can be divided into two dimensions. The first is monitoring, where it supervises, controls the operation, and analyses the system in real-time. The other is tracking the variations in the behavior of a target, which may be a person, animal, or event [12].

WSNs plays a significant role when they are deployed in the environment. In the forest, they detect the behavior of wildlife, wildfire outbreaks, and landslides, in addition to monitoring and tracking air pollution, earthquakes, flood detection, and many other disasters [13,14]. In agriculture, WSNs can be used in smart farming, crop management, irrigation management, disease detection, and yield prediction, as well as monitoring temperature, humidity, and soil moisture measurements [15,16]. In the medical field, WSNs can be used for remote patient status monitoring, diagnosis, and emergency response [17]. They can also be used in other healthy applications, such as eating habits, fitness issues, and monitoring sportive activities [18]. In transportation, WSNs can be used in parking lot management, railway tracks, and management systems, as well as monitoring traffic and roads [19]. In military and national security applications, WSNs plays a vital role [20]. The widespread application of WSNs has led to the existence of the Internet of Things (IoT), which depends on sensory devices that detect the information and send it through the internet to be processed and make decisions for the ease, safety, and comfort of human life. In the IoT, WSNs are applied in smart homes, health, agriculture, industry, building, city, grids, and much more [21].

### 2.4. Wireless Sensor Network Constraints

Even though WSNs are used in many applications, there are several constraints and challenges that face WSN deployment, coverage, and connectivity. Some of these factors are [22]:

- Limited energy resources: Due to the small size of a sensor node, the battery supported will be small, with a limited lifetime, and this leads to limited processing power and limited storage capacity, resulting in increasing the energy consumption problem.
- Low data rate: There is a higher latency in WSN communication. WSN works in short communication ranges, and the transmission data rate depends on the frequency used.
- Communication failures: The failed nodes result in communication failures, so there should be a fault tolerance to overcome the interruptions when this occurs.
- Security issues: The wireless communication channels of WSN are vulnerable to passive and active attacks, thus resulting in serious problems.

### 2.5. Deployment in Wireless Sensor Network

The factor that has an important effect on all the WSN performance metrics is deployment. The optimal deployment of the sensor nodes indicates that the whole required area is covered, and the network nodes have the best communication with each other with a minimal number of nodes [23]. Deployment can be either static or dynamic; static deployment is divided into deterministic and random deployment, depending on the environment and application required [24].

#### 2.5.1. Static Deployment

**Figure 5:**
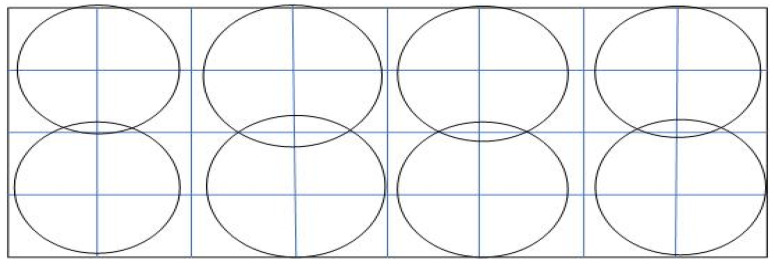
Deterministic deployment.

When the information regarding the application area is known in advance or the sensor node position can be determined, then the deterministic deployment will be applied as shown in Figure 5. Most of the research studies that depend on deterministic deployment set up the node location based on geometrical structure. This can be in a two-dimensional plane, such as a square, triangular, hexagon, and tri-hexagon tiling grids; it was proven that the regular hexagon is the best topology in the two-dimensional plane [25]. The other deployment can be in three-dimensional space by using the three-dimensional mathematical model and space geometric theory to find a solution for the coverage problem in many three-dimensional applications, which can be classified as binary and probabilistic coverage models. The space geometric methods can be divided into the volumetric quotient-based approach and spherical overlap approach (k-coverage) [26].

#### 2.5.2. Random Deployment

**Figure 6:**
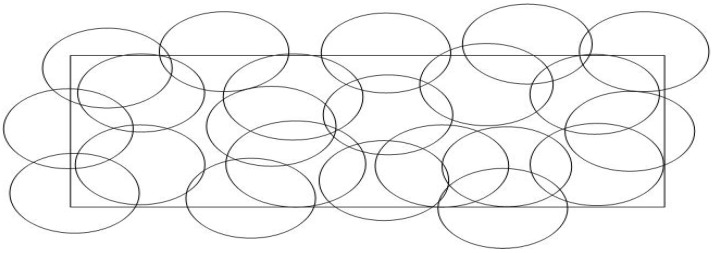
Random deployment.

When the sensing area is difficult to reach (to put sensors) or not known in advance, due to disasters, fire forests, or battlefields, then the random deployment is preferred, i.e., randomly dispersing the nodes in the desired area, which can be thrown from a plane in a disaster area, for example. This method is simple but, at the same time, has many drawbacks, such as bad coverage or loss of connectivity due to obstacles or failures. So many optimization strategies are used to find the best location, taking the requirements of coverage, connectivity, lifetime, and robustness into account to achieve one of these objectives or some of them simultaneously. Classical, heuristic, and meta-heuristic optimization algorithms are used to solve the random deployment problem of WSNs. Random deployment can be represented in Figure 6.

#### 2.5.3. Dynamic Deployment

**Figure 7:**
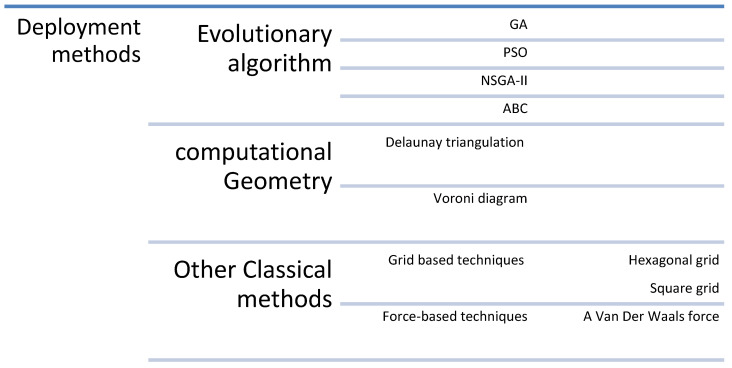
Deployment methods.

Dynamic deployment is used with mobile WSNs, when the objective is to monitor an event or increase the network’s coverage, connectivity, and lifetime issues. In dynamic deployment or self-deployment, the nodes are randomly deployed first; then, the nodes change their locations to increase the coverage, which means each point in the area of interest should be covered. This type of deployment can be either centralized or distributed. In centralized deployment, the sink node should be a powerful node because it is responsible for finding the new location of each sensor node through deployment algorithms and sending the required location to each sensor. This type of deployment saves energy but may fall to single-point failure problems and cannot be implemented in disasters or battlefields. While the distributed deployment lets each node determines its new position in the monitoring area, this may consume more energy [27]. A summary of deployment methods is shown in Figure 7.

### 2.6. Coverage and Connectivity in WSN

Two essential issues in WSNs are coverage and connectivity. If the sensor node is active and can detect an object in the monitored region, then we can say that this object is covered by the WSN. Coverage can be divided into three types, i.e., area, point, and barrier coverage [28,29]. Area coverage can be full or partial coverage; full coverage means that each point in the monitoring area should be covered by at least one sensor node or K (where K ≥ 1) node, according to the application requirements, such as a battlefield, where it is necessary to have precise information about the observed area. Other applications, such as environment applications, may require partial coverage. Partial coverage can be useful when the number of sensor nodes is not enough for full coverage. This can maximize network lifetime and save energy.

In point coverage, a specific point or target should be monitored, which may be static or mobile, such as observing the behavior of an animal in the region of interest or monitoring some important points on the enemy battlefield. Barrier coverage means monitoring international borders and trying to detect any illegal behavior. Additionally, barrier coverage can be full or partial coverage; sensors are deployed based on the Poisson point access model in full and partial coverage and used with a limited number of sensors.

Concerning connectivity, the network is said to be fully connected if each sensor node has at least one path to the sink node to transmit and receive data. If there is full coverage without connectivity, the WSN quality of service will be degraded. So, these two issues, i.e., coverage and connectivity, should be always considered simultaneously in the deployment of WSNs. In some applications that required full coverage, full connectivity is also required to achieve data gathering and transmitting to the sink node. Two types of connectivity are available, i.e., one-connectivity when there is a single path between the sensor and sink nodes and k-connectivity when there are multiple paths from the sensor node to the sink node.

**Figure 8:**
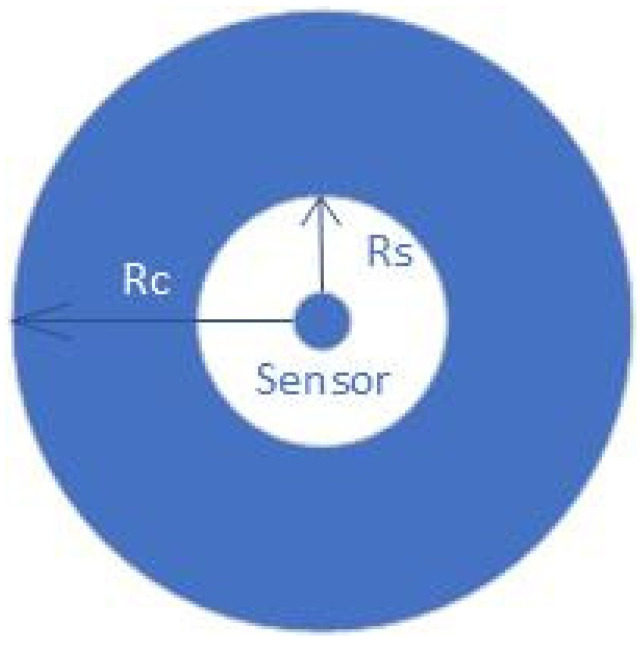
Sensing range Rs and communication range Rc.

There is always a relation between coverage and connectivity for each application. It is almost assumed that the range of connectivity (Rc) is greater than, or twice, the sensing range (Rs), as shown in Figure 8 [25,30].

If we assume that there is a point P located at the (x, y) coordinates and sensor node Si located at (xi, yi) coordinates, then the Euclidean distance between point P and the sensor node Si can be expressed as follows [31,32]:

(1) $$d\left(\mathrm{Si},P\right)=\sqrt{{\left(\mathrm{xi}-x\right)}^{2}+{\left(\mathrm{yi}-y\right)}^{2}}$$

The general sensibility of a sensor node on point P can be expressed as follows:

(2) (Si, P)=δ(d(Si, P))k

where **δ** and **k** represent the non-negative, sensor-dependent constants.

There is an inverse relationship between the sensor sensitivity and Euclidean distance between the sensor node and monitored point in the region of interest—as the distance increased, the sensitivity decreased.

**Figure 9:**
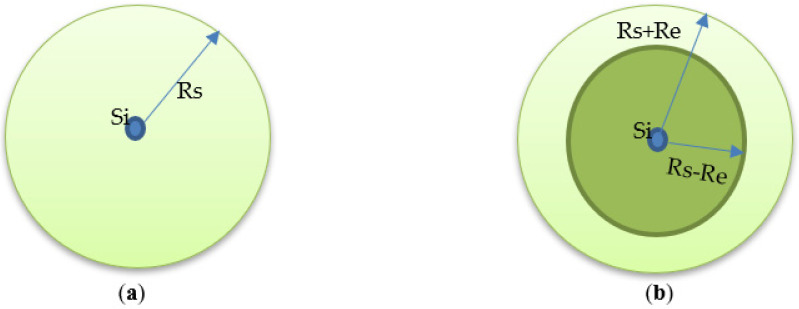
Sensing models. (**a**) Binary model. (**b**) Probabilistic model.

**Tow sensing models are found**:

**Binary Disk sensing model**: The simplest sensing model is the binary sensing model, which is represented in Figure 9a. When point P lies within the sensing radius of the sensor node Rs, then this point is covered by the sensor node—otherwise not. The coverage equation can be expressed as follows:

(3) $$C_{\mathrm{xy}}\left(\mathrm{Si}\right)=\{\begin{array}{ll} 1 & \mathrm{if}\;d\left(\mathrm{Si},\;P\right)<\mathrm{Rs}\\ 0 & \mathrm{other}\;\mathrm{wise} \end{array}$$

**Probabilistic sensing model**: This model is more practical and comprehensive than the binary model; it assumes that the sensed event, sensor design, and environmental conditions are all stochastic in nature. The coverage equation can be expressed as follows:

(4) Cxy(Si)={0,if Rs + Re ≤ d(Si,P)e−δαβif Rs − Re <d(Si,P)<Rs+Re1,if Rs−Re≥d(Si,P)

where

**Re** is the uncertainty measure in sensor detecon, 0 < Re < Rs,

a = d(Si,P) − (Rs − Re);

**δ** and **β** are parameters that measure the detection probability when the target in a distance equal to

Rs − Re < d(Si,P) < Rs + Re.

The probabilistic sensing model is represented in Figure 9b.

## 3. Literature Review

Numerous strategies are used in the literature to solve the deployment problem of WSNs for optimizing coverage and connectivity issues. One of them is optimization algorithms, which can be single- or multiple-objective; the single-objective optimization algorithm is not suitable for real applications, since optimizing one performance metric may inversely affect another metric; for example, in sensor node deployment, maximizing coverage needs large spread number of sensor nodes, and this will increase the consumed energy, hence reducing the network lifetime. For this reason, the solution goes towards using the multi-objective optimization algorithms in WSN deployment, which aims to satisfy multiple goals at the same time, with a set of constraints, which will be a real challenge. Meta-heuristic search algorithms have been widely used in this area, since they can provide multiple elements in the Pareto front in a single evaluation, as they have a population-based nature. These algorithms have the advantages of preventing the local optimum traps and reaching the global optimum points [33]. Within this line of research, Saad et al. (2020) proposed an improved multi-objective genetic algorithm NSGA-II (non-dominated sorting genetic algorithm II) to implement a new suggested mathematical formula for 3D WSNS deployment problem with directional sensing ability, in order to maximize the coverage and minimize the deployment cost. Through extensive simulation, they proved the performance of the proposed formula, while using the proposed Bresenham line-of-sight coverage model and assuming real sensors and 3D environment models [34].

Khaoula, Z. et al. (2020) [35] suggested a conceptual framework to maximize the sensing coverage, and lifetime and minimize the total deployment cost of WSN in the smart building by using the building information modeling (BIM) database, which includes all the required information about the building and uses the sensor parameters as input data to the proposed system; then, an evolutionary algorithm (genetic algorithm) will be used to solve the optimization problem. After that, this optimized solution will be visualized using the BIM plugin tool in an off-line and real-time 3D building model, considering heterogeneous sensors and different obstacles. The decision variable vector of the optimization problem will be the sensor node location in the smart building, while the constraint is to find at least one path between the sink node and each sensor node, in order to construct a connected graph.

Due to the emergence of 3D data regarding urban terrain, Bin C et al. (2019) [36] used this data to implement a heterogeneous wireless directional sensor network deployment in smart cities by optimizing three objectives, i.e., coverage, connectivity quality, and lifetime, while simultaneously considering connectivity and reliability as a constraint. They used a graph-based 3D SPM signal propagation model, which evolved by employing a line-of-sight (LOS) model for simulating wireless signals and calculating the path loss and intensity of the signal at a given point. To solve the optimization problem, they proposed a distributed parallel multi-objective evolutionary algorithm (MOEAs), with a message passing interface (MPI) called a distributed parallel cooperative coevolutionary multi-objective large-scale evolutionary algorithm with multiple populations (DPCCMOLSEA-MP). Two types of terrain data were used, i.e., even and rough. This proposed algorithm was compared with other evolutionary algorithms, in terms of performance and operation time.

Another optimization algorithm called the flower pollination algorithm was modified by Zhendong, W. et al. (2019) [37] to propose two new versions of the algorithm. The first is called the improved flower pollination algorithm (IFPL), which is a single-objective optimization algorithm used to maximize the coverage area of WSN deployment in an urban area, assuming for solar batteries and heterogeneous sensors with obstacles. This modification includes the use of tent chaotic mapping to generate the mapping sequence, as well as the use of a nonlinear convergence factor, so that the convergence ability of the algorithm can be improved. Additionally, greedy cross-over strategy was used to enhance the accuracy of the solution.

The second proposed version of the algorithm, called the non-dominated sorting multi-objective flower pollination algorithm (NSMOFPA), is a multi-objective optimization algorithm used to maximize the coverage rate, thus minimizing the energy consumption and node radiation overflow rates, assuming for heterogeneous sensor nodes that are deployed in forest environment having non-rechargeable batteries with obstacles. The global pollination problem is solved by using the external archive and leader strategies, and the diversity of the population can be maintained by using the proposed crowding degree method and elite strategy. These two proposed algorithms are applied to the WSN deployment problem, through extensive simulation experiments using the MATLAB 2014b program. After comparing with other optimization algorithms, they found that IFPA can provide enhanced in-network coverage and deployment cost. Additionally, the NSMOFPA has a better optimization solution for WSN deployment.

Smart parking is one of the smart city applications. Slimane Ch et al. (2021) [38] developed a new optimization algorithm for WSN system deployment for fire detection in smart car parks called multi-objective binary integer linear programing (MOBILP). The nodes in these networks are divided into two groups, i.e., sensor nodes for target monitoring and relay nodes that receive alert messages from the sensor nodes. This proposed algorithm aims to simultaneously minimize the number of sensor and relay nodes, as well as decrease the maximum distance between sensor and sink nodes, while ensuring coverage and connectivity. They evaluate this method through different tests and compare the results with existing work, such as the mono-objective function.

Energy consumption and high cost are the disadvantages that present when deploying homogenous or heterogeneous WSNs to solve coverage problems. So, Belal et al. (2020) developed a deployment model that depends on the probability sensing model (PSM) and harmony search algorithm (HAS) to attain the balance between the coverage performance and cost of heterogeneous WSN. PSM is used to solve the overlapping problem between nodes. The proposed model is evaluated through multiple simulation scenarios, using MATLAB, by analyzing the coverage ratio and cost and comparing the obtained results with those from the scenarios that used a homogenous model and meta-heuristic algorithm, such as the genetic algorithm [39].

WSN lifetimes depends on energy consumption and the covered area. To have efficient coverage and energy usage, there must be an optimum network deployment because it affects all network performance. Aparajita et al. (2021) proposed an optimization deployment algorithm that used glowworm swarm optimization, K-means algorithm, and Voronoi cell structure for optimizing coverage and energy consumption with a minimum number of nodes, multi-hop transmission, and sleep-wake mechanism [40]. To increase network lifetimes, network clustering is used, which means dividing the network into virtual groups. For each group there is a cluster head, with powerful capabilities, that is responsible for gathering data and sending it to the base station, either directly or through multi-hope routing. Cluster performance is a very important issue, since it affects network lifetimes. Mohit, K. et al. (2021) proposed a modified genetic algorithm-based load-balanced clustering algorithm for WSN (MGALBC) that depends on residual energy. Then, compare it with the (GALBC) algorithm. The new suggested algorithm shows an enhancement in network lifetime, energy consumption, and the number of active sensor nodes [41].

Ahmed et al. (2021) [42] tried to enhance WSN coverage and cost by proposing a multi-objective optimization algorithm (MOO) with variable-length decision space for sensor deployment in a 2D environment. This algorithm used social class multi-objective particle swarm optimization with V-length nature (SC-MOPSO). It expands the concept of social interaction of particle swarm optimization by dividing the solution space into classes according to their dimensions and combines inter- and intra-class operators, in order to confirm the required dynamics of the solution changes to satisfy the Pareto front. This algorithm was evaluated through a different experiment by comparing it with weighted sum variable length particle swarm optimization (WS-VLPSO) and nondominated sorting genetic algorithm (NSGA-II).

One of the WSN applications is border surveillance. Amira, Z. et al. (2021) conducted a real experiment on the Tunisia–Libya border. The aim was to achieve full coverage and connectivity through the deterministic deployment of sensor nodes. They took the parameters of sensor density, consumed energy, and quality of sensor coverage into account when trying to reach k-coverage and connectivity with a minimum number of sensor nodes. Different types of WSNs were used in this experiment, i.e., were the wireless multimedia sensor and marine wireless sensor networks, as border monitoring systems [36].

Kalaipriyan, T. et al. (2021) [43] proposed an optimization deployment algorithm for the target wireless sensor network (T-WSN) based on an evolutionary-based non-dominated sorting genetic algorithm (NSGA-II) to solve a multi-objective problem of increasing coverage and connectivity for the target monitoring. Pseudo-codes were written, and multiple scenarios were implemented using the MATLAB simulation tool. The non-dominated sorting keeps the better solutions in multiple objectives simultaneously using dominant relation. The performance evaluation of this multi-objective algorithm was performed in terms of performance indicators, i.e., overall non-dominated vector generation (ONGV) and spacing (SP). This algorithm showed the best performance, after comparison with other algorithms.

Considering the mobility, environment properties and using heterogeneous nodes in the WSN increases its deployment problem complexity. Fatima H. et al. (2021) [44] took these problems into account when designing their optimization deployment algorithm based on integer linear programming (ILP). The objective of this algorithm is to maximize coverage, while taking network lifetime, mobility, and heterogeneity as constraints. They observed and discussed the importance of subarea monitoring in this research. For large-scale monitoring areas, they proposed using a swarm intelligence meta-heuristic algorithm for network deployment. In a simulation experiment, they evaluated this algorithm by comparing the coverage ratio and energy consumption with other recent algorithms. While Mohsen Sh. et al. (2021) [45] solved the problem of target and area coverage and connectivity in randomly distributed homogeneous and heterogeneous WSNs, considering both centralized and distributed nodes, using the steepest descent (SD) analytical deployment algorithm with Armojo and Wolf rules, instead of evolutionary algorithms. The proposed method was compared with the genetic algorithm, in the case of moving the sensor nodes towards the target. Through simulations, they found that it outperforms the genetic algorithm; however, in the case of considering both coverage and connectivity, besides managing sensor movement in the required area, a hybrid algorithm was used, which consisted first of the genetic algorithm to define the first positions of the sensor nodes and then using the steepest descent algorithm to move these sensors to the optimal locations. They found that this method can support the sensor’s trajectory and better accuracy for network coverage and connectivity.

To solve the energy-efficient coverage problem for randomly deployed mobile WSN with obstacles, Pakarat M. et al. (2022) [46] produced an improved competitive swarm optimizer to increase the covered area and decrease energy consumption at the same time. They also used the virtual force algorithm (VFA) and Voronoi diagram (VD) to enhance network performance through the optimization process. To control the position of the sensors, the VFA was integrated with a boundary mechanism, while the VD was used to get the network information for the decoding process.

## 4. Research Method

In this paper, we applied a systematic mapping method to conduct the search. For a particular area, it supports an overview and result summary of published papers from the answers to research questions and classification of studies. The most important advantage is to define gaps in the existing research, which provides new topics to investigate [47].

**Figure 10:**
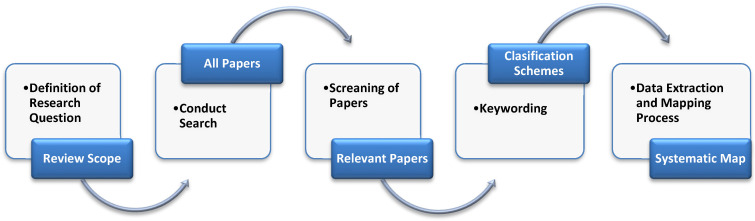
Systematic mapping process.

The mapping study process, shown in Figure 10, consisted of five steps: identify the research question; gather the search in the sources; chose the papers that answer the research question; classify the papers; and find data and map studies to finalize the data classification and summarization.

### 4.1. Research Questions

In this study, the following research questions (RQs) have been identified and discussed. Each research question is related to a particular aspect of WSN deployment optimization in smart cities, as listed below:

1. What is the number and distribution of studies published on WSN deployment optimization in the period between 2015–2022?
2. Which are the most used optimization algorithms in the current studies that are related to WSN deployment optimization in smart cities?
3. What are the advantages of using optimization algorithms in solving the deployment problem of WSN?
4. What are the performance metrics that should be considered when deploying WSNs in smart cities?
5. What are the most used simulation and software platforms to simulate and analyze the WSN deployment scenarios in the literature?
6. What are the challenges and issues that WSN deployment is facing in smart cities?
7. What are the potential future issues for WSN deployment in smart cities?

### 4.2. Scientific Databases and Search Strategy

Four online academic search engines were used to conduct the search and find the relevant papers:

1. IEEE Xplore digital library;
2. Science direct;
3. Springer link;
4. Scopus.

To make an automatic search on the chosen libraries, the search string consisted of the following sections:

**Wireless**, **sensor**, **network**, (**deployment**, **deployment algorithm**, or **deployment optimization**), and **smart city**.

In the screening phase, the papers that were initially collected were filtered until only papers that answer the research questions remained. In this work, relevant papers were selected using the following inclusion and exclusion criterion.

**Inclusion criterion:**

- Publications related directly to the deployment of WSN sensor nodes using optimization algorithms.
- Publications dealing with enhancing or maximizing the coverage and connectivity of WSNs.
- Publications in the field of WSN deployment in smart city applications.

**Exclusion criterion**:

- Papers published before 2015.
- Publications not written in the English language.
- Publications related to other types of WSN deployment methods, such as using geometric or classical deployment methods.
- Publications related to other WSN problems, such as localization, routing, data gathering, etc.

Starting with the general search string “WSN deployment”, the number of publications collected from IEEE Xplore digital library was 253, Science Direct was 354, SpringerLink was 621, and Scopus was 586 documents. After using inclusion and exclusion criterion and removing duplications, the total number of relevant studies was 68 documents that related directly to the deployment problem of WSN using optimization algorithms during this period.

## 5. Results

Mapping studies are often carried out based solely on the abstracts. The primary study selection was increased by applying the search criteria to all of the following sections: title, abstract, introduction, and conclusion. The included papers, as well as a comparison between their used algorithms and objectives, are listed in Table 1. The following subsections show the results and discussions of each research question.

### 5.1. Distribution of Studies (RQ1)

**Figure 11:**
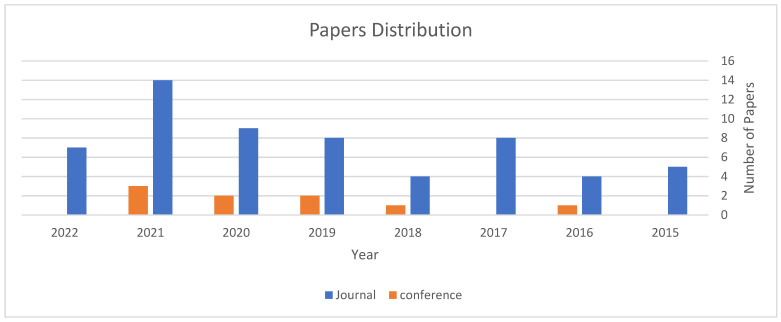
Papers distribution.

**Figure 12:**
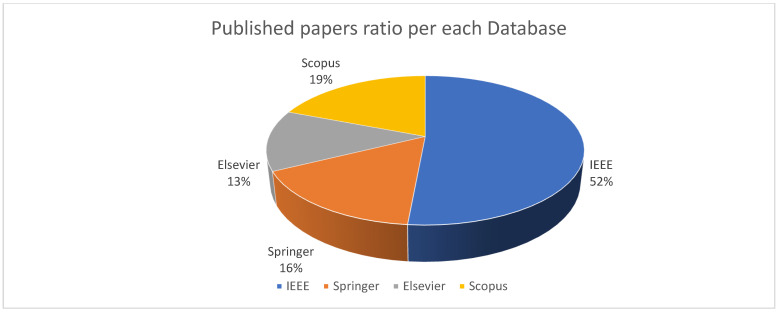
Papers ratio for each database.

The selected papers were analyzed to find their number and distribution, as belonging to the period 2015–2022, as shown in Figure 11. It is clear that the number of studies that deal with this topic increased each year, since there is an incremental usage of smart city applications around the world. A total of 35 of the selected papers were published in the IEEE journals and conferences, while 11 papers were published in Springer, 9 in Elsevier, and 13 in other Scopus journals. The ratio of these numbers to the total number of the chosen papers is shown in Figure 12.

### 5.2. Optimization Algorithms (RQ2 &RQ3)

Most of the reviewed studies used meta-heuristic optimization algorithms to solve the deployment problem. The meta-heuristic stands for the Greek words meta and *heuriskein*, which means solving problems using an upper-level methodology. Meta-heuristic algorithms represent a part of optimization in computer science and applied mathematics concerning algorithms and computational complexity theory, which depend on inspiring their solutions from natural habits, such as particle swarms, annealing processes, and ant colonies. These types of algorithms are used in many fields, such as artificial intelligence, mathematical programming, soft computing, and operations research. These algorithms are general approximate algorithms that can be deployed to different types of optimization problems and can be updated to solve any hard problem, since they provide fast, flexible, and robust solutions; besides, they are easy to design and implement. The disadvantage of these algorithms is that there is no guarantee that the approximated solution is closed to the optimal solution. Further, because of the no free lunch (NFL) theorem, there is no meta-heuristic algorithm that can be used generally for different optimization problems [103]. This means that an optimization algorithm can outperform well for a specific problem but not so well with another one. Hence, there is always a need for newly proposed optimization algorithms to find solutions for more complex problems. This can be done either by proposing new algorithms or updating existing ones or by a combination of two different types of optimization algorithms, such as a hybrid algorithm between classical and meta-heuristic algorithms or meta-heuristic and artificial intelligence algorithms [11].

**Figure 13:**
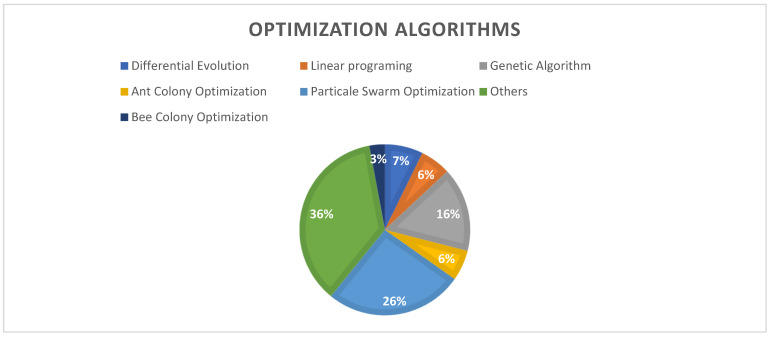
Optimization algorithms ratios.

About 35% of the reviewed studies worked on an update to swarm intelligence optimization algorithms [40,44,46] such as particle swarm optimization (PSO) [42,58,66,68,69,74,75,76,77,81,88,97,99], ant colony optimization (ACO) [33], and bee colony optimization (BCO) [48,65], due to their ability to solve complex problems and provide a satisfactory solution in a feasible time [90]. These algorithms are applied to enhance network performance by combining them with other approaches and then comparing the obtained results with other algorithms, such as the genetic, greedy, and multi-objective evolutionary algorithms. The ratio of participation for each algorithm is shown in Figure 13.

### 5.3. Performance Metrics (RQ4)

Many performance parameters or metrics should be considered when deploying WSNs, starting with coverage and connectivity, which are the most important parameters that ensure high quality of service from WSNs, as discussed earlier in Section 2.6, as well as other parameters, such as the lifetime, energy consumption, latency, signal strength (RSSI), accuracy, scalability, reliability, and more [32]. A brief discussion of these parameters is explained below.

• **Lifetime**

One of the most important metrics in WSNs is the network’s lifetime. Through research, it is found that network lifetime depends on two dimensions, the first is network connectivity, which means that network lifetime can be defined as the period from the network deployment until one or more nodes lost connection to the sink node. While the second dimension depends on energy consumption, and this means network lifetime is the period from network deployment until one or multiple live nodes fall below a specific energy threshold [104]. Most conducted studies in Table 1 tried to maximize lifetime through optimal node deployment that maximizes network coverage and connectivity, hence minimizing the power consumption that leads to prolonged network lifetime.

• **Energy Consumption**

Each node in WSN needs energy in three parts, i.e., collecting, processing, and communicating data. The amount of consumed energy depends not only on the energy capacity but also on the heterogenous functionalities of the sensor node. The efficient use of energy prolongs the network lifetime. This can be achieved through reducing the number of exchanged messages between nodes and scheduling sleep intervals for redundant nodes, while leaving the remaining nodes active to save network coverage and connectivity, as well as using efficient routing protocols; all these steps will minimize energy consumption [28].

• **Latency and accuracy**

Latency and accuracy are related to each other. Latency means the time required to send a message from the source node to the destination node across the network, and this represents the total delay of the sent message. Accuracy represents the efficient arrival of the sent message to the destination within the limited time required, so reducing delay ensures network accuracy, and this is an important performance metric in WSN [11].

• **Signal strength (RSSI)**

This metric represents a measure of link quality and depends on the distance between two nodes to calculate the reachability of the node through the communication process. RSSI stands for received signal strength indicator that can be determined from the following equation [76,105]:

RSSI = −10 × n × log_10_(d) + p (5)

where,

d: is the distance from the sensor node measured in meters;
n: is the propagation constant or path-loss exponent;
p: is the power in reception mode (Dbm) (decibel-milliwatts).

• **Scalability and Reliability**

Scalability can be defined as the ability of the network to be extended by including more nodes in the network, while preserving network performance. The reliability of a WSN represents its ability to deliver sensed data to the sink node, while maintaining coverage and connectivity through the mission period. This period is application-dependent [90].

While using optimization algorithms in solving the WSN deployment problem, the performance metrics that have been presented in the literature to measure the qualities of the approximations collected from different optimization algorithms can be described as follows [95].

• **The number of non-dominated solutions (NDS)**

If Ps represents the set that contains all the non-dominated solutions generated by the proposed algorithm A, then the NDS-metric is defined by the size of Ps, as follows:

(6) $$\mathrm{NDS}=\left|P_{s}\right|$$

A higher value of NDS means that a sufficient number of choices exist [95].

• **Set coverage metric (C-metric)**

If there are two optimal sets of non-dominated solutions, then the set coverage is the comparison metric between these two sets, which represents the ratio of the non-dominated solutions in set_2_, dominated by non-dominated solutions in set_1_ and divided by the number of solutions in the set_2_. This means, if C(set_1_,set_2_) < C(set_2_,set_1_), then set_2_ has better a solution than set_1_ [99].

• **Diversity metric (Δ)**

The diversity metric (Δ) determines the range of spread accomplished through the obtained solutions.

• **Hypervolume metric (HV-metric or S-metric)**

The hypervolume metric (HV) (also defined as the S-metric) presents the joint information about closeness and diversity in the acquired non-dominated set of solutions (Ps) [42].

• **Generational distance metric (GD-metric)**

The generational distance metric (GD-metric) determines how far the acquired Ps set of solutions is from the true set PT.

• **Computation time or complexity**

The computation time of the optimization algorithm is required to measure its efficiency. An efficient algorithm should provide optimal solutions within an acceptable time. So, this metric is important for comparison between the algorithms [43,46].

The performance metrics of each conducted study in this survey are presented in Table 1.

### 5.4. Simulation Programs (RQ5)

The preferable simulation tool for WSN deployment using an optimization algorithm is the MATLAB/Simulink program [106,107], which was used by many researchers in the literature, as illustrated in Table 1. This efficient simulation program was designed by MathWorks. MATLAB stands for matrix laboratory used with Simulink tools to design, simulate, and analyze embedded systems. It contains some toolboxes to generate new network scenarios, and the simulation data can be visualized using five tools, which are the MATLAB graphics, port value display, scopes, dashboard block library, and simulation data inspector. This simulation program can work in two modes, the deterministic and probabilistic modes. The first mode is used for code testing and debugging, while the second one is used for wireless communication. It can be used to simulate MAC layer operation, radio transmission, and collision detection in ad hoc networks for any number of sensor nodes via an embedded tool called Powerler.

### 5.5. Challenges, Limitations, and Future Issues for WSN Deployment in Smart Cities (RQ5&RQ6)

Node deployment of WSN can be either indoor or outdoor, depending on the smart city application requirements. Indoor deployment can be applied in closed areas, such as buildings and structures, while outdoor deployment can be applied in open and harsh areas, such as roads, gardens, forests, and volcanoes. So, the area of the monitored region plays a significant role in determining the deployment type to be random, deterministic, or dynamic [108]. The network coverage type also depends on the nature of the monitored area, which can be area, target, or barrier coverage. In addition, some applications require one-connectivity, that is, a single path between sensor and sink node; others require more reliable connectivity, called k-connectivity, with the sink node [109].

Each study has its own advantages, disadvantages, and limitations, according to the application, environment, sensing model, coverage type, and required objectives, but it has been observed that most of these have the following issues:

- Most of the research studies deal with homogenous nodes in WSN and use unreal and simplified models.
- Most of the research studies deal with 2D plane deployment, while modern applications require 3D space deployment.
- Security, reliability, scalability, and energy consumption are also important issues that need to be considered with coverage and connectivity when deploying a sensor node [110,111].
- Most papers do not take the existence of the obstacle into account when determining coverage and connectivity.
- Localization techniques need to be merged with the deployment techniques, in order to increase reliability and robustness.

In the future, most of these limitations should be taken into account when designing an optimization deployment algorithm for WSN node deployment.

## 6. Conclusions

This paper provides a systematic mapping study regarding the deployment problems of WSN sensor nodes in smart city applications using the meta-heuristic optimization method. First, a detailed background on smart city and WSN is presented; then, the earlier studies on WSN deployment are reviewed. The research methodology discussed the research questions and used databases and inclusion and exclusion criterion to obtain the results. It was found that 68 papers in the period between 2015–2022 related directly to the WSN deployment using meta-heuristic optimization algorithms, and 35% of these studies focused on updating the swarm optimization methods. Most of the selected studies used the MATLAB simulation tool to design and code the WSN optimization algorithm for its efficiency and simplicity. Finally, this paper discussed the challenges and limitations that WSN deployment faces in smart cities, as well as suggested future issues.

## Figures and Tables

**Table 1:** Comparison between recent studies that discuss the deployment problem.

Paper	Application	Space	Methodology and Simulation Tool	Objective(s)	Performance Metrics
Pakarat, M. et al. (2022) [46]	Open area	2D	Competitive swarm optimizer, virtual force algorithm, and Voronoi diagram	Maximize coverage for mobile WSN and minimize the energy consumption simultaneously	Coverage ratio Moving distance Average sensing radius Dissipated energy coverage convergence curve
Sathian, D. et al. (2022) [48]	Smart farming	2D	Artificial bee colony-based, energy-efficient, multiple-input, multiple-output routing protocol, MATLAB R2018b simulation tool	Minimize the network cost by minimizing the number of deployed sensor nodes; maximizing network lifetime	Lifetime Energy utilization Throughput Packet loss
Adnan, T. et al. (2022) [49]	Open area	2D	Immune plasma algorithm	Maximize coverage, and lifetime and minimize consuming energy	Coverage ratio
Yindi, Y. et al. (2022) [50]	Remote environmental monitoring	2D	Improved moth flame search	Repair coverage holes and minimize energy consumption	Coverage rate Maximum moving distance Average moving distance Coverage efficiency
Qin, W. et al. (2022) [51]	Harsh environment	2D	Vampire bat algorithm and improved virtual force, MATLAB 2016b simulation tool	Repair coverage holes and minimize energy consumption	Coverage rate Moving distance Complexity
Yin-Di, Y. et al. (2022) [52]	Remote monitoring	2D	Discrete army ant search optimizer, MATLAB 2016a simulation tool	Maximizing target coverage	Coverage ratio
Nour El-Houda, B. et al. (2022) [53]	Indoor environment	2D	Improved multi-objective Evolutionary algorithm, case study	Enhancing network quality of service	Execution time Cost Coverage rate Connectivity
Slimane Ch et al. (2021) [38]	Fire detection in a smart car park	2D	Multi-objective binary integer linear programing	Simultaneously minimize the number of sensors and relay nodes, besides decreasing the maximum distance between sensor and sink node, while ensuring coverage and connectivity	Complexity Running time Cost
Aparajita et al. (2021) [40]	Randomly deployed dynamic networks	2D	Glowworm swarm optimization, K-means algorithm, and Voronoi cell structure, MATLAB 2017 a	Optimizing coverage and energy consumption, with a minimum number of nodes, multi-hop transmission, and sleep-wake mechanisms	Coverage rate
Ahmed et al. (2021) [42]	Any environment	2D	Social class multi-objective particle swarm Optimization with V-length nature	Enhance WSN coverage and cost	Set coverage Number of nondominated solutions Hypervolume Delta metric
Amira, Z. et al. (2021) [54]	Border surveillance	2D	Deterministic deployment	Achieve full coverage and connectivity	K-coverage Connectivity
Kalaipriyan, T. et al. (2021) [43]	Target monitoring	2D	Evolutionary-based non-dominated sorting genetic algorithm, MATLAB 8.4	Increasing coverage and connectivity for target monitoring	F-value Computational time
Fatima, H. et al. (2021) [44]	Subarea and large-scale area monitoring.	2D	Integer linear programming and swarm intelligence meta-heuristic algorithm, MATLAB	Maximize coverage, while taking network lifetime, mobility, and heterogeneity as constraints	Lifetime Coverage ratio
Mohsen, Sh et al. (2021) [45]	Target and area monitoring	2D	Steepest descent analytical deployment algorithm with Armojo and Wolf rules. MATLAB	Maximize coverage and connectivity	Target coverage Connectivity Area coverage rate
Kavita, J. et al. (2021) [55]	Smart IoT applications	2D	Grey wolf-based optimization technique, MATLAB R2018b simulation tool	Maximizing coverage and connectivity and minimizing overall network cost	Coverage Connectivity Cost Time complexity and scalability
Fan, Y. et al. (2021) [56]	Mixed-crop farmlands	2D	The greedy algorithm, MATLAB R2018b simulation tool	Maximizing coverage and connectivity and reducing deployment costs;	Cost Overlap rate
Chun-Han, H. et al. (2021) [57]	Open area	2D	Self-economic for single-objective real parameter optimization problem, C++ programming language	maximizing the coverage rate of all the targets, while minimizing the energy consumption of the static and mobile sensors	Lifetime Evaluation number
Xiaogang, Q. et al. (2021) [58]	Open area	2D	Embedded virtual force resampling particle swarm optimization algorithm, MATLAB 2018	Coverage improvement	Coverage rate
Chandra, N. et al. (2021) [59]	Open area	2D	Biogeography-based optimization, MATLAB 2018a	Maximize coverage, minimize the number of sensor nodes, and minimize interference with efficient connectivity	Sensing interference rate Target point coverage rate Selection of potential position rate
Fang, F. et al. (2021) [60]	Square area	2D	A parallel version of the sine cosine algorithm	Enhance dynamic sensor node distribution	Convergence rate Coverage rate
Onat, G. et al. (2021) [61]	Indoor placement	3D	Multi-objective integer linear programming model, YALMIP (MATLAB optimization toolbox)	Maximize coverage and system robustness	Robustness rate Coverage rate
Li-Gang, Z. et al. (2021) [62]	Terrain coverage	3D	Hybrid algorithm depends on shuffled frog leaping algorithm and whale optimization algorithm, CEC2017 test set	Improve network coverage with a minimum number of nodes	Convergence rate
Li, C. et al. (2021) [63]	Open areas	2D	Social spider optimization algorithm, MATLAB R2017	Improve network coverage and cost	Convergence ability Coverage effect Connectivity Reliability Energy consumption Simulation time
Junbin, L. et al. (2021) [64]	Pipeline monitoring	2D	Submodular optimization algorithm, EPANET, and MATLAB.	Maximize monitoring capacity of large-scale pipeline network	Monitoring capacity Number of mobile sensors Computing time
Salah, B. et al. (2020) [31]	Area monitoring	2D	Multi-objective genetic algorithm and the weighted sum optimization method, Python	Ensure coverage, connectivity, and cost	Topology k-coverage ratio m-connectivity ratio Sensing Probability
A. Saad et al. (2020) [34]	Terrain topology	3D	An improved multi-objective genetic algorithm	Maximize the coverage and minimize the deployment cost	Execution time Coverage rate Number of deployed sensors
Khaoula, Z. et al. (2020) [35]	smart building	3D	Building information modeling database and genetic algorithm	Maximize the sensing coverage and lifetime and minimize the total deployment cost of WSN	Coverage Network lifetime Cost Connectivity Number of sensor nodes
Belal et al. (2020) [39]	Urban area	2D	Probability sensing model and harmony search algorithm, MATLAB	Attain the balance between the coverage performance and cost of heterogeneous WSNs; PSM was used to solve the overlapping problem between nodes	Coverage Cost
Puri, V. et al. (2020) [65]	Target monitoring	2D	Hybridizes the artificial Bee colony and whale optimization algorithms, MATLAB	Maximize coverage and connectivity	Coverage rate Connectivity rate
Yanzhi, D. (2020) [66]	Area monitoring	3D	combined the distributed particle swarm Optimization algorithm and a proposed 3D virtual force algorithm, MATLAB (R2016a)	Maximize coverage and maintain connectivity	Connectivity ratio Lifetime Coverage ratio
Zhendong, W. et al. (2020) [67]	Area monitoring	3D	Enhanced grey wolf optimizer, MATLAB 2014b	Improve WSN coverage and save deployment cost	Convergence Time complexity Coverage rate Network connectivity
Weiqiang, W. (2020) [68]	Smart cities	2D	Adaptive particle swarm optimization algorithm, OMNET++5.0, MATLAB2014a	Improving network QoS	Convergence trajectory Secure connectivity rate
Wang Y, (2020) [69]	Dairy farming	2D	Particle swarm optimization, MATLAB	Improve network coverage and connectivity	Coverage rate
Na, X. et al. (2020) [70]	Field monitoring	2D	Discrete particle swarm optimization	Improved field monitoring	Detectability Convergence speed Scalability
Ramin, Y. et al. (2020) [71]	Target monitoring	2D	Cooperative particle swarm optimization and cooperative particle swarm optimization using fuzzy logic, C++	Prolonging the network lifetime	Network lifetime Number of deployed sensors
Beyza, G. et al. (2019) [33]	Dynamic deployment	2D	Quick ant bee colony, c-sharp programing language, net framework 4.6.1	Improved network performance	Convergence rate CPU time
Bin, C. et al. (2019) [36]	smart cities	3D	Multi-objective evolutionary algorithm with message passing interface	Optimizing coverage, connectivity quality, and lifetime, while simultaneously considering connectivity and reliability as a constraints	Operation time hypervolume (HV) indicator
Zhendong, W. et al. (2019) [37]	Urban areas Forest areas	2D	Improved flower pollination algorithm non-dominated sorting multi-objective flower pollination algorithm, MATLAB 2014b	Maximize the coverage area of WSN deployment in an urban area Maximize the coverage rate, minimize the energy consumption rate, and minimize the node radiation overflow rate	Time complexity Population convergence Coverage rate Pareto front
Yamin, H. et al. (2019) [72]	Area coverage	2D	Improved differential evolution	Maximize coverage	Coverage rate Convergence speed
Faten, H. et al. (2019) [73]	Area monitoring	2D	Multi-objective flower pollination algorithm	Enhance coverage, reduce energy consumption, maximize lifetime, and maintain connectivity	Energy consumption lifetime
Hongshan, K. (2019) [74]	Area coverage	2D	Enhanced practical swarm optimization	Maximize coverage	Coverage rate
Tripatjot, S. et al. (2019) [75]	Area coverage	2D	Hybrid technique practical swarm optimization + Hooke–Jeeves search method	Maximize coverage	Coverage rate
Zhanjun, H. et al. (2019) [76]	Area coverage	3D	Improved practical swarm optimization, real experiment (RSSI)	Maximize coverage	Coverage rate Received signal strength indicator (RSSI)
Vishal, P. et al. (2019) [77]	Target coverage	2D	Genetic algorithm and practical swarm optimization, MATLAB	Improve coverage and connectivity	Moving distance
Yung, P. et al. (2019) [78]	Environment monitoring	3D	Kmeans embedded in genetic algorithm, MATLAB2014b	Reduced deployment time and cost	Generational distance Number of solutions in Pareto optimal set Number of sensors and relay nodes Execution time
Wei, L. et al. (2018) [79]	Area coverage	2D	Ant-lion optimization algorithm, MATLAB R2016a	Increase coverage rate	Coverage rate
Yongquan, Z et al. (2018) [80]	Area coverage	2D	Social spider algorithm, MATLAB 2012a	Improve coverage	Coverage rate Convergence speed Complexity
Aparna, P et al. (2018) [81]	Area coverage	2D	Modified discrete binary particle swarm optimization	Improve coverage	Normalized overhead Packets dropped Throughput Lifetime
Tehreem, Q. et al. (2018) [82]	Environment monitoring	3D	Ant colony optimization, MATLAB	Improve network performance	Computational cost Number of deployed sensor nodes
Bin, C. et al. (2018) [83]	Terrain monitoring	3D	Modified directional evolution algorithm	Considering network coverage, connectivity, and lifetime of sensor node	Fitness value Operation time
Hossein, M. et al. (2017) [84]	Area coverage	2D	Multi-objective optimization evolutionary algorithm based on decomposition	Improve coverage, power consumption, delay, reliability, and lifetime	Connectivity Coverage Reliability Lifetime
Ozan, Z. et al. (2017) [85]	Area coverage	2D	Modified genetic algorithm	Coverage improvement	Coverage rate
Enes, A. et al. (2017) [86]	Area coverage	2D	K-means for clustering and simulated annealing for deployment optimization, python	Maximize coverage and reduce deployment cost	Confusion and Accuracy Coverage priority
Shu-Yu, K. et al. (2017) [87]	Surveillance application	2D	Quantum-inspired tabu search algorithm with entanglement, C++	Improve coverage and connectivity	Computational complexity Connectivity Coverage rate
Qingjian, N. et al. (2017) [88]	Area coverage	2D	Heterogeneous multi-swarm practical swarm optimization	Improve coverage and reduce energy consumption	Coverage rate Fitness value
Yasser El K et al. (2017) [89]	Area coverage Barrier coverage	2D	Hybridize gradient method and the simulated annealing algorithm, MATLAB	Achieve full coverage with minimum number of nodes	Coverage rate CPU time
Dina, S. et al. (2017) [90]	IoT application	2D	Ant colony optimization+ local search	Improve reliability	Success rate of feasible solutions Number of deployed sensors
Xiaojian, Z. et al. (2017) [91]	Target coverage	2D	Compare greedy heuristic, local search, and practical swarm optimization, Java programming	Satisfy coverage quality requirement	Success rate Network deployment cost Running time
Osama, M. et al. (2017) [92]	Field monitoring	2D	Harmony search, MATLAB	Maximize coverage and minimize cost	Minimum distance between sensors Coverage rate Sensing range and cell size
A. Xenakis et al. (2016) [93]	Area coverage	2D	Simulated annealing	Maximize coverage and minimize energy consumption	Coverage rate Consuming energy
Ahmed, B. et al. (2016) [94]	Air quality monitoring	2D	Integer programming model-enhanced atmospheric dispersion simulator called SIRANE	Enhance the quality of pollution estimation with minimum cost	Coverage cost
Mina Kh. Et al. (2016) [95]	Area coverage	2D	Constrained Pareto-based multi-objective evolutionary approach, MATLAB	Maximize coverage, minimize energy consumption, prolong the lifetime, and maintain connectivity	Number of non-dominated solutions Set coverage Diversity Hypervolume Generational distance Computation time Coverage Lifetime
Mustapha, R. et al. (2016) [96]	Surveillance application	2D	Genetic algorithm, ANSI-C++	Maximize detection rate and minimize false alarm rate	Running time Number of deployed sensors Deployment cost Coverage rate
Aparna, P. et al. (2016) [97]	Area coverage	2D	Modified discrete binary practical swarm optimization, NS3.21	Improve coverage	Number of iterations Convergence Time
Liu, C. et al. (2015) [98]	Structural health monitoring (SHM)	3D	Genetic algorithm (GA)	Improve energy consumption and modal identification accuracy	Energy consumption Accuracy Number of deployed sensors
Matthieu Le. et al. (2015) [99]	Target tracking	2D	Non-dominated sorting genetic algorithm-II, multi-objective practical swarm optimization, specific heuristic (H3P), C++	Improve coverage, minimize sensor node number and non-accuracy	Coverage of two Pareto fronts (C metric) The proportion of optimal solutions
Danping, H. et al. (2015) [100]	Indoor and outdoor application	3D	Multi-objective genetic algorithm, C++	Optimize network performance	Maximum number of generations Population size Evolutionary possibilities Computation time Received signal strength Coverage Connectivity Cost Lifetime Energy consumption Packet latency Packet drop rate
Junfeng, C. et al. (2015) [101]	Area coverage	2D	Brainstorm optimization, K-means for clustering, MATLAB 8.0	Improve coverage	Coverage rate
Pooja, N. et al. (2015) [102]	Area coverage	2D	Bacteria foraging	Improve coverage and connectivity	Coverage rate
